# Cardiotonic Pills Plus Recombinant Human Prourokinase Ameliorates Atherosclerotic Lesions in LDLR^–/–^ Mice

**DOI:** 10.3389/fphys.2019.01128

**Published:** 2019-09-10

**Authors:** Jing-Na Deng, Quan Li, Kai Sun, Chun-Shui Pan, Huan Li, Jing-Yu Fan, Gao Li, Bai-He Hu, Xin Chang, Jing-Yan Han

**Affiliations:** ^1^Department of Integration of Chinese and Western Medicine, School of Basic Medical Sciences, Peking University, Beijing, China; ^2^Tasly Microcirculation Research Center, Peking University Health Science Center, Beijing, China; ^3^State Key Laboratory of Bioactive Substances and Functions of Natural Medicines, Institute of Materia Medica, Chinese Academy of Medical Sciences and Peking Union Medical College, Beijing, China; ^4^Key Laboratory of Microcirculation, State Administration of Traditional Chinese Medicine of the People’s Republic of China, Beijing, China; ^5^Key Laboratory of Stasis and Phlegm, State Administration of Traditional Chinese Medicine of the People’s Republic of China, Beijing, China; ^6^State Key Laboratory of Core Technology in Innovative Chinese Medicine, Tianjin, China; ^7^Department of Oncology, Guizhou University of Chinese Medicine, Guiyang, China

**Keywords:** atherosclerosis, inflammation, ATP-binding cassette gene 8, adipose triglyceride lipase, scavenger receptor A, CD36

## Abstract

**Aim:**

This study was to explore the protective effects of cardiotonic pills (CP) or/and recombinant human prourokinase (proUK)on the atherosclerosis and the potential underlying mechanism.

**Methods and Results:**

Atherosclerosis was induced in LDLR^–^/^–^ mice by high fat diet contained 20% lard and 0.5% cholesterol. Daily oral administration of CP (130 mg/kg) or/and intravenous injection of proUK (2.5 mg/kg, twice a week) began at 8 weeks after feeding with high fat diet and continued for 4 weeks. CP alone treatment markedly decreased plasma triglyceride, but did not ameliorate atherosclerosis plaque. No effect was observed for proUK alone on any endpoints tested. CP plus proUK induced a significantly reduction in the atherosclerotic lesions, along with decreased levels of total cholesterol, triglyceride in the plasma. CP plus proUK inhibited the elevated hepatic total cholesterol and triglyceride in high fat diet-fed LDLR^–/–^ mice, up-regulating the expressions of ATP-binding cassette gene 5 and 8, and adipose triglyceride lipase. In the aorta, CP plus proUK inhibited the expression of scavenger receptor A and CD36 in LDLR^–/–^ mice. In addition, we observed that systemic inflammation was inhibited, manifested downregulation of plasma macrophage inflammatory protein-1α and intercellular cell adhesion molecule-1.

**Conclusion:**

CP plus proUK effectively attenuated atherosclerosis plaque in LDLR^–/–^ mice, which is associated with normalizing the lipid metabolism in the liver and aorta, reducing phagocytosis of receptor-mediated modified-LDL uptake and inhibiting systemic inflammation.

## Introduction

Atherosclerosis (AS) is a chronic disease of the arterial wall which is the leading cause of disability and death around the world (Castelli et al., 1986; Pant et al., 2014). Hyperlipidemia, system inflammation and hypertensionare thought to be the major risk factors in the formation and development of AS (Zhu et al., 2018).

Lipids play a central role in the pathogenesis of AS. Plasma low-density lipoprotein (LDL) levels have a positive association with the development of AS. AS is initiated by endothelial dysfunction predominantly due to the accumulation of plasma LDL (Alonso et al., 2014). Endothelial cell dysfunction leads to the infiltration of LDL particles and their subsequent oxidation to oxidized-LDL (ox-LDL). Macrophages differentiated from circulating monocyte take up ox-LDL via scavenger receptor A(SR-A) and CD36 to form foam cell (Lusis, 2000; Zhu et al., 2018).

Liver is the major organ that regulates plasma lipids balance especially the LDL content. Disrupted hepatic total cholesterol (TC) and triglyceride (TG) homeostasis contributes to the pathogenesis of dyslipidemia, hepatic lipid deposition and atherosclerosis (Kim et al., 2014). There are two ways to decrease hepatic cholesterol: reducing cholesterol synthesis or excreting excess cholesterol into bile by reverse cholesterol transportation (Hageman et al., 2010). It has been reported repeatedly that increasing the turnover of bile salts has a beneficial effect on LDL-C levels. Several bile salt sequestrates have been successfully used to reduce LDL-C levels in patients with hypercholesterolemia (Maxfield and Tabas, 2005). Hepatic TG deposition is manifested as increased lipogenesis, disrupted fatty acid (FA) oxidation and depressed triglyceride (TG) lipolysis. Hormone sensitive lipase (HSL) and adipose triglyceride lipase (ATGL) are two enzymes critical for hepatic neutral cholesterol ester hydrolase (Buers et al., 2009; Buchebner et al., 2010). ATGL^–/–^ macrophages accumulate TG-rich lipid droplets resulting in altered cell morphology that resemble macrophage foam cells. These alterations and functional changes strongly argue for the involvement of ATGL in atherogenesis. Transplantation of ATGL^–/–^ bone marrow into γ-irradiated LDLR^–/–^ mice resulted in highly attenuated atherosclerotic lesion formation compared with WT bone marrow-transplanted LDLR^–/–^ mice after feeding high fat diet for 9 weeks (Paul et al., 2008; Radovic et al., 2012).

Inflammation plays an important role in the initiation and progression of atherosclerotic plaque. Inflammatory signaling alters the behavior of the intrinsic cells of the artery wall (endothelium and smooth muscle), and recruits further inflammatory cells that interact to promote lesion formation and complications. In humans, ongoing inflammatory reactions within the coronary atherosclerotic plaques are increasingly thought to be crucial determinants of the clinical course of patients with coronary artery disease (Stoll and Bendszus, 2006).

The cardiotonic pill (CP) is a compound Chinese medicine composed of *Salvia miltiorrhiza, Panax notoginseng*, and *Borneol*, which has been widely and effectively used in cardiovascular diseases in China. The major active components of CP are dihydroxy-phenyl lactic acid, tanshinone II-A (both from *S. miltiorrhiza*), and notoginsenoside R1 (from *P. notoginseng*). Our previous studies revealed that CP attenuated myocardial I/R injury, protecting against microcirculatory disturbance, cardiac dysfunction, and myocardium infarction. CP protected against post infarction myocardial fibrosis along with a reduction in chemokine production, macrophage infiltration, and fibroblast activation (Wei et al., 2013; Yang et al., 2013). Recent reports suggested that CP normalized hyperlipimedia, improving vascular function and other pathological processes (Niu et al., 2000; Zhao et al., 2010; Guo et al., 2016; Han et al., 2017; Lim et al., 2017). Recombinant human prourokinase (proUK) (Geng et al., 2018; Hao et al., 2018; Zhao et al., 2018) is a novel type of thrombolytic drug for clinical application in thrombotic diseases, which selectively activates fibrinogen on the surface of the thrombus, and has less side effect of bleeding (Sasahara et al., 1995; Verstraete, 1999). However, no study is reported as to the effect of CP or proUK on progression of atherosclerosis. The present study is to evaluate the effect of CP or/and proUK on the atherosclerosis and the potential underlying mechanism.

## Materials and Methods

### Materials

Cardiotonic pills (Batch no. 150203) and proUK (Batch no. 20170501) were supplied by Tasly Pharmaceutical Co., Ltd. (Tianjin, China). The processing of the product followed a strict quality control, and the ingredients were subjected to standardization.

Antibodies recognizing ATGL and GAPDH were from Cell Signaling Technology (Boston, MA, United States). Antibodies against CD36, SR-A, SR-BI, PPARα, ABCA1, ABCG1, ABCG5, andABCG8 were from Abcam (Cambridge, MA, United States). Oil Red O was from Sigma-Aldrich (St. Louis, MO, United States). All other reagents used in our study were of analytical grade.

### Animals

LDLR^–/–^ mice were purchased from Animals Center of Peking University Health Science Center. Animals were raised at a temperature of 20 ± 2°C with 12-h light/dark cycles, and fed with standard rat chow and water. All surgical procedures performed on animals were approved by Peking University Biomedical Ethics Committee Experimental Animal Ethics Branch (LA2010001). Procedures involving animals and their care were conducted in conformity with international and national law and policies (EU Directive 2010/63/EU for animal experiments, ARRIVE guidelines and the Basel declaration including the 3R concept).

Male 6-8-weeks-old LDLR^–/–^ mice were acclimated to our animal care facilities for 5 days before experiment, with free access to normal chow diet and water. Animals were first randomly allocated to two groups: chow diet (*n* = 8) and high fat diet (*n* = 102). Animals in high fat diet group were fed with high fat diet contained 20% lard, 0.5% cholesterol, 79.5% basic diet. After 8 weeks, the high fat diet-fed LDLR−/− mice were randomly divided into five groups: high fat diet + NS (model, *n* = 20), high fat diet + CP (*n* = 28), high fat diet + proUK (*n* = 20), high fat diet + CP plus proUK (*n* = 26), high fat diet + Atorvastatin plus Aspirin (*n* = 8). CP was administrated at 130 mg/kg daily by gavage, atorvastatin and aspirin also were intragastrically administered at doses of 6 and 15 mg/kg/day, respectively. ProUK was intravenously injected at 2.5 mg/kg, twice a week, for 4 weeks. CP, atorvastatin and aspirin were dissolved in purified water and used at a clinical equivalent dose. ProUK was diluted with physiologic saline and administrated at a quarter of the clinical dose. The model group mice were given the same volume of purified water and saline. Body weight and plasma lipids content was measured every 2 weeks.

### En Face and Histology Analyses

LDLR^–/–^ mice were sacrificed and rinsed with 20 ml phosphate buffered saline (PBS) followed by perfusion with 4% paraformaldehyde in PBS through the left ventricle. Aortas were fixed in 4% paraformaldehyde overnight, and then kept in 20% sucrose at 4°C until they sank. The aortas were then stained with Oil Red O after stripping off connective tissues and adipose tissue (Zhao et al., 2015). The en face aortic lesions were observed using a Canon EOS 80D digital camera and quantified using Image-Pro Plus 6.0 (Media Cybernetics Inc., MD, United States) analysis and expressed as percent lesion area.

Sections of the aortic tissues were harvested, embedded in optimal cutting temperature compound and then frozen. Series sections of 7-μm-thick of frozen aortic tissue were collected beginning at the aortic sinus. Every fifth section was stained with Oil Red O and counterstained with Hematoxylin and eosin (HE) as routine. Atherosclerotic lesion areas were measured using Image-Pro Plus 6.0 and expressed as average Oil Red O staining area per section in the first seven sections for each mouse.

### Ultrastructure Analyses

At the end of experiment, the LDLR^–/–^ mouse liver (*n* = 3 for each group) was perfused for 30 min with 3% glutaraldehyde (Ted Pella, Redding, CA, United States) in 0.1 mol/L PBS at a speed of 3 mL/min. For transmission electron microscopy, a slice approximate 1 mm thick was taken and stored in freshly prepared 3% glutaraldehyde overnight at 4°C. After rinsing with 0.1 mol/L PBS for 3 times, the tissue block was post-fixed in 1% osmium tetroxide in 0.1 mol/L PBS for 2 h at 4°C. The samples were dehydrated and then embedded in Epon 812 (SPI-CHEM, Westchester, PA, United States). Ultra-thin sections of liver were stained with uranium acetate and lead citrate and examined in a transmission electron microscope (JEM-1400 Plus, JEOL, Tokyo, Japan).

### Experiments in Cultured Macrophages

RAW264.7 macrophages were cultured in a high glucose DMEM medium containing 10% fetal calf serum, in which 50 μg/ml ox-LDL or 50 μg/ml Dil ox-LDL (Yuabio, Beijing, China) was added. The cells were incubated with or without CP, proUK, or CP plus proUK for 24 h. The final concentrations of CP and proUK were 0.5 mg/ml, 10 μg/ml, respectively. The contents of total cholesterol (TC), and triglyceride (TG) were tested by commercial kits from Applygen Technologies (Beijing, China). The cells incubated with Dil ox-LDL were observed under a Nikon Eclipse 50i microscope.

### ELISA and Flow Cytometer Analyses

After LDLR^–/–^ mice were fasted for 6 h, blood samples were collected from heart. The samples were centrifuged at 500 g for 20 min at 4°C to separate plasma. Plasma TC and TG contents, HDL-C and LDL-C levels were detected with kits from BioSino Bio-technology and Science Inc. (Beijing, China). Plasma AST, ALT and ICAM-1 levels were measured by enzyme-linked immunosorbent assay using an insulin ultrasensitive ELISA kit (R&D, Minneapolis, MN, United States), according the instruction of the manufacture. The color absorbance at 450 nm was measured using a Bio-Rad microplate reader.

Plasma cytokines were determined by BD Cytometric Bead Array with a flow cytometer (FACS Calibur, BD, Franklin Lakes, NJ, United States) at the end of experiments as described previously (Zhang et al., 2014; Mao et al., 2015). The following cytokines were determined in this study using their corresponding antibody: IL-1α, IL-1β, IL-6, IL-10, TNF-α, MCP-1, and MIP-1 α (R&D Systems, Minneapolis, MN, United States).

### Western Blotting Analyses

Liver or aorta tissues were lysed in sample buffer containing 62 mM Tris–HCl, pH 6.8, 0.1% SDS, 0.1 mM sodium orthovanadate, and 50 mM sodium fluoride. The protein content was determined by the BCA protein assay (Applygen Technologies Inc., Beijing, China). Equal amount of proteins was loaded and separated by SDS-PAGE. After electrophoresis, the proteins were transferred on membranes, after being blocked with 3% non-fat dry milk, the membrane with target proteins was recognized with primary antibodies against CD36, SR-A, SR-BI, PPARα, ABCA1, ABCG1, ABCG5, ABCG8, and GAPDH (Abcam, Cambridge, MA, United States). The bands were detected using an ECL detection kit (Applygen Technologies, Beijing, China). For quantification, band intensity was assessed by densitometry and expressed as mean area density using Quantity One image analyzer software (Bio-Rad, Richmond, CA, United States).

### Statistical Analysis

All data were expressed as mean ± SEM. Statistical analysis was performed using one-way ANOVA followed by a Tukey *post hoc* test. A value of *P* < 0.05 was considered statistically significant.

## Results

### CP Plus proUK Slows Down the Progression of Atherosclerosis Plaque in LDLR^–/–^ Mice

**FIGURE 1:**
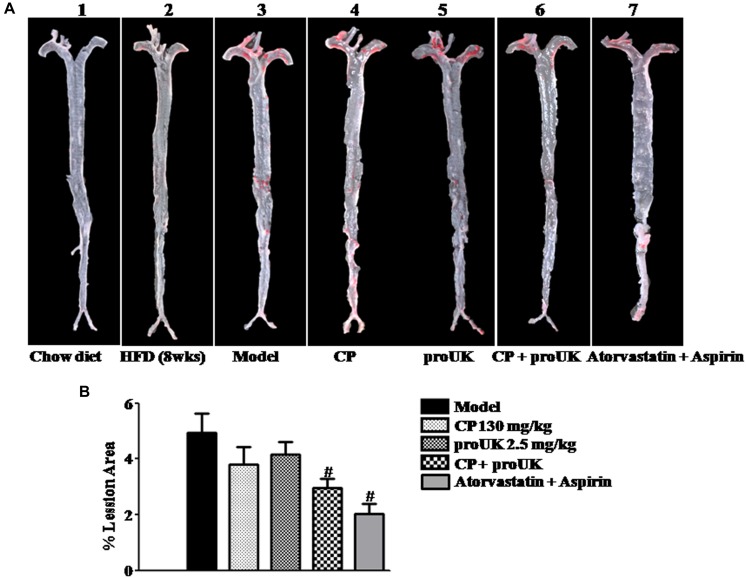
CP plus proUK inhibits the development of atherosclerosis in LDLR^–/–^ mice. High fat diet-fed LDLR^–/–^ mice were randomly divided into 4 groups and received CP orally or/and proUK injection. **(A)** Representative images of en face Oil Red O staining of the full length of aorta of LDLR^–^/^–^ mice from Chow diet group **(A-1)**, *n* = 3. High fat diet-fed mice for 8 weeks group **(A-2)**, *n* = 2. Model group **(A-3)**, *n* = 12. CP + HFD group **(A-4)**, *n* = 12. proUK + HFD group **(A-5)**, *n* = 12. CP plus proUK group **(A-6)**, *n* = 12. Atorvastatin + Aspirin group **(A-7)**, *n* = 8. **(B)** Percent lesion area of aorta in different groups. Data are presented as the percentages of total en face aortic area. *n* = 10. ^#^*P* < 0.05 vs. Model mice.

**FIGURE 2:**
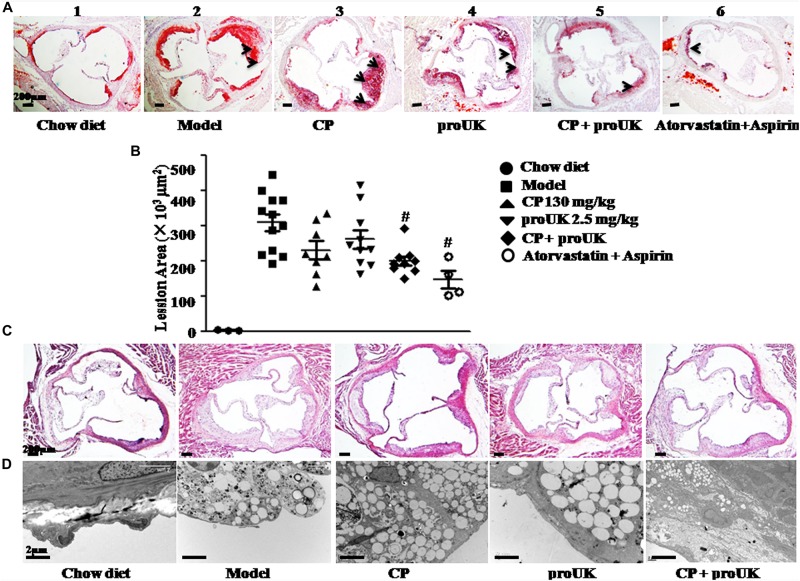
CP plus proUK reduces atherosclerosis lesion area in aortic root of LDLR^–/–^ mice. At the end of the treatment, aortic roots were prepared for transmission electron microscopy or as 7-μm frozen cross sections and stained with HE or Oil Red O solution. **(A)** Oil Red O staining of the left ventricular outflow tract in LDLR^–/–^ mice from Chow diet group **(A-1)**, *n* = 3, Model group **(A-2)**, *n* = 10, CP + HFD group **(A-3)**, *n* = 8, proUK + HFD group **(A-4)**, *n* = 10, CP + proUK group **(A-5)**, *n* = 8. Atorvastatin + Aspirin group **(A-6)**, *n* = 4, Bar = 200 μm. The arrow shows the atherosclerosis plaque. **(B)** The statistic results of Oil Red O staining atherosclerosis lesions. *n* = 8–10. ^#^*P* < 0.05 vs. Model mice. **(C)** HE staining of the left ventricular outflow tract in LDLR^–^/^–^ mice from Chow diet group **(B-1)**, Model group **(B-2)**, CP + HFD group **(B-3)**, proUK + HFD group **(B-4)**, CP + proUK group **(B-5)**. Bar = 200 μm. **(D)** Transmission electron microscopic images of the aortic arch in LDLR^–/–^ mice from Chow diet group **(C-1)**, Model group **(C-2)**, CP + HFD group **(C-3)**, proUK + HFD group **(C-4)**, CP + proUK group **(C-5)**. Bar = 2 μm.

**FIGURE 3:**
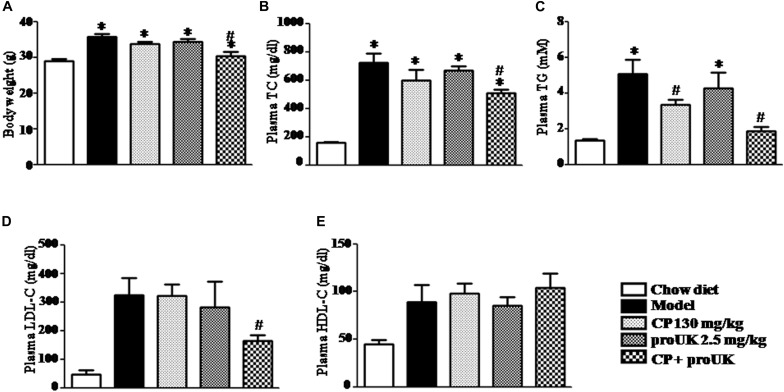
CP plus proUK attenuates the increase in body weight and plasma TG, TC and LDL-C in the high fat diet-induced mice. **(A)** The body weight at the end of experiment. **(B)** Plasma TC level in different groups mice. **(C)** Plasma TG level in different groups mice. **(D)** Plasma LDL-C level in different groups mice. **(E)** Plasma HDL-C level in different groups mice. Total cholesterol, triglycerides, LDL-c, and HDL-c were quantified by enzymatic assays. The values are presented as the means SEM. *n* = 10. ^∗^*P* < 0.05 vs. Chow diet mice, ^#^*P* < 0.05 vs. Model mice.

**FIGURE 4:**
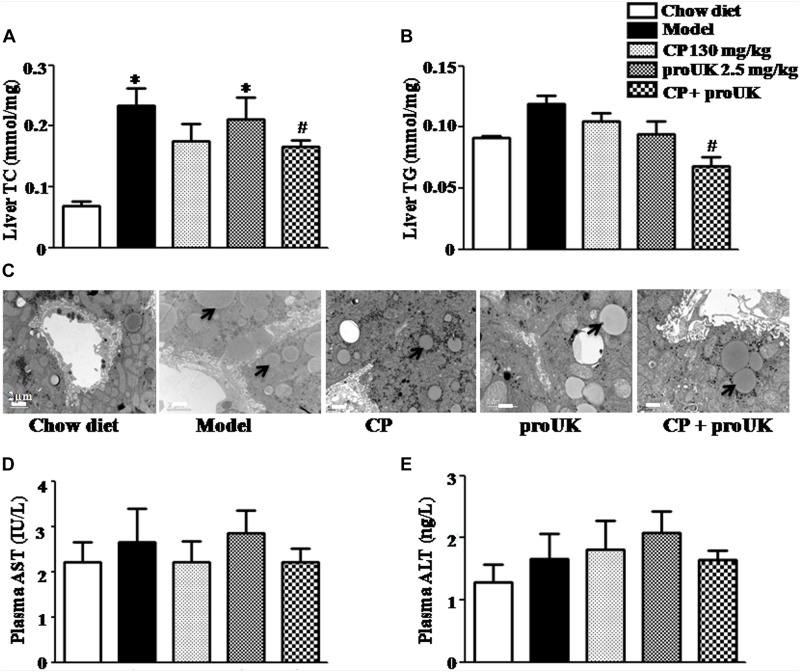
CP plus proUK attenuates hepatic lipid deposition and increase in TC and TG while does not affect liver function in high fat diet-fed LDLR^–/–^ mice. **(A)** Hepatic TG levels in different groups. **(B)** Hepatic TC levels in different groups. **(C)** Transmission electron microscopic images of the sinusoids in different groups. Bar = 2 μm. **(D)** AST activity in plasma from different groups. **(E)** ALT activity in plasma from different group. Data are mean ± SEM (*n* = 8). The arrow shows the lipid droplet. ^∗^*P* < 0.05 vs. Chow diet mice, ^#^*P* < 0.05 vs. Model mice.

**FIGURE 5:**
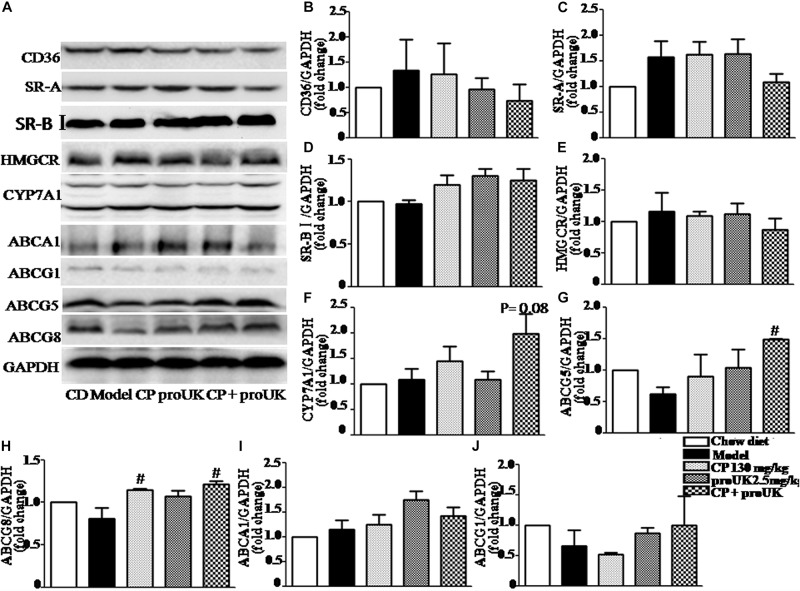
Effect of CP plus proUK on the proteins associated with lipid metabolism in the livers of LDLR^–^/^–^ mice. **(A)** Western blotting of CD36, SR-A, SR-BI, ABCA1, ABCG1, ABCG5, ABCG8, HMGCR, and CYP7A1 in the livers of LDLR^–^/^–^ mice from different groups. **(B–D)** The quantitative analysis of the expression of scavenger receptor CD36, SR-A, SR-BI in the liver from different groups. **(E)** The quantitative analysis of the expression of cholesterol synthesis rate-limiting enzyme HMGCR in the liver from different groups. **(F)** The quantitative analysis of CYP7A1 expression in the liver from different groups. **(G)** The quantitation of the ratio betweenABCG5 protein/GAPDH protein in the liver from different groups. **(H)** The quantitation of the ratio betweenABCG8 protein/GAPDH protein in the liver from different groups. **(I)** The quantitative analysis of ABCA1 expression in the liver from different groups. **(J)** The quantitative analysis of ABCG1 expression in the liver from different groups. *n* = 5. ^#^*P* < 0.05 vs. Model mice.

**FIGURE 6:**
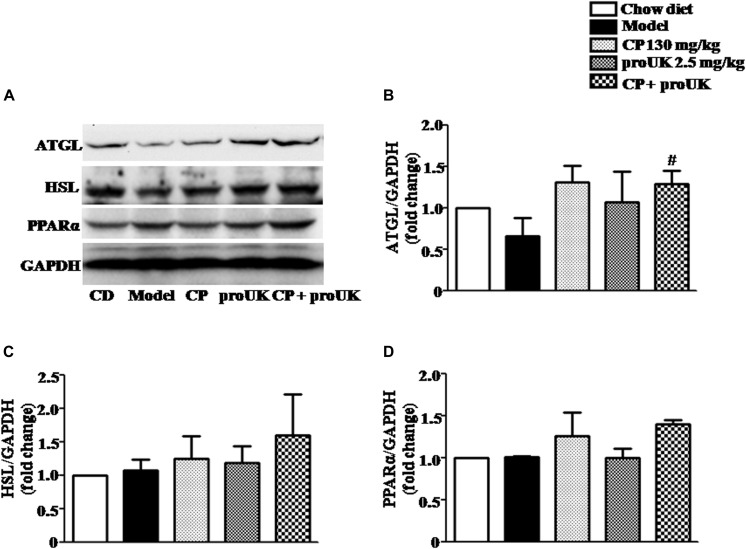
Effect of treatment with CP plus proUK on the expression of hepatic triglyceride metabolism-related proteins in the liver. **(A)** Western blot of the liver protein levels of ATGL, HSL and PPARα. **(B)** The quantitative analysis of ATGL expression in the liver from different groups. **(C)** The quantitative analysis of HSL expression in the liver from different groups. **(D)** The quantitative analysis of PPARα expression in the liver from different groups. Data are presented as the mean ± SEM. *n* = 5. ^#^*P* < 0.05 vs. Model mice.

**FIGURE 7:**
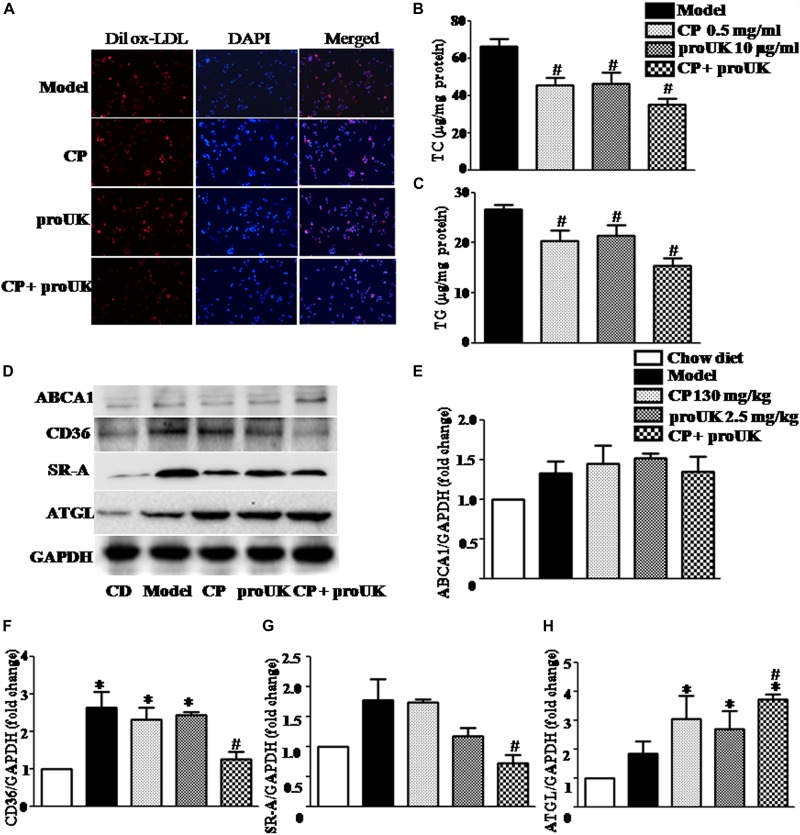
CP plus proUK decreases the accumulation of lipids in RAW264.7 macrophages and the levels of CD36 and SRA in the aorta of LDLR^–/–^ mice. **(A)** The accumulation of lipid droplets in RAW264.7 macrophages. **(B)** The effect of CP or/with proUK on the level of TC in RAW264.7 macrophages. **(C)** The effect of CP or/with proUK on the level of TG in RAW264.7 macrophages. **(D)** Western blot of ABCA1, CD36, SRA1 and ATGL from the aorta in LDLR^–/–^ mice from different groups. **(E)** The quantitative analysis of ABCA1 expression in aorta from different groups. **(F)** The quantitative analysis of CD36 expression in aorta from different groups. **(G)** The quantitative analysis of SR-A expression in aorta from different groups. **(H)** The quantitative analysis of ATGL expression in aorta from different groups. Data are presented as the mean ± SEM. *n* = 4. ^∗^*P* < 0.05 vs. Chow diet mice, ^#^*P* < 0.05 vs. Model mice.

The initial atherosclerotic plaque formed at the active arch after 8 weeks of high fat diet induction (Figure 1A-2) but not in chow diet mice (Figure 1A-1). To investigate the role of CP or/and proUK in atherosclerotic plaque formation, we compared aortic lesion in different groups after 4 weeks of treatment. Aortic en face Oil Red O staining showed that mice treated with CP plus proUK (Figure 1A-6) developed less atherosclerotic lesions than model mice (Figure 1A-3). The quantification of en face aortas revealed a significant reduction (36.0%) in lesion sizes in CP plus proUK-treated mice (Figure 1B). Treatment with CP (Figure 1A-4) or proUK (Figure 1A-5) alone did not significantly ameliorate atherosclerotic plaques compared to model mice. As a positive control, Figure 1A-7 revealed that treatment with 6 mg/kg atorvastatin plus 15 mg/kg aspirin for 4 weeks significantly inhibited the development of high fat-diet induced atherosclerotic plaques in LDLR^–/–^ mice.

The results in Figure 1 indicate that most of the lesions are presented in the aortic arch. We further stained the frozen slices of the aortic sinus with Oil Red O (Figure 2A) and HE (Figure 2C). Compared to model mice (Figure 2A-2), mice treated with CP plus proUK (Figure 2A-5) revealed reduced lesion areas by 34.8% (Figure 2D), while no significant decrease in lesion area was found in CP (Figure 2A-3) or proUK (Figure 2A-4) alone treated group. As a positive control, the plaque area of the aortic sinus was less prominent in treatment with atorvastatin plus aspirin group compared with model group (Figure 2A-6). Consistent with the result of Oil Red O staining, HE staining (Figure 2C) and transmission electron microscopy (Figure 2D) results showed that the accumulation of foam cells was abated in the aortic sinus from CP plus proUK treated LDLR^–/–^ mice compared with model mice.

Taken together, the results in Figures 1, 2 suggest that CP plus proUK had better protective effect on plaque formation than either of the two alone.

### CP Plus proUK Reduces the Body Weight and Plasma Lipids in High Fat Diet-Fed LDLR^–/–^ Mice

High fat diet resulted in a significantly increase in body weight (Figure 3A), as well as in the plasma level of TC, TG, LDL-C, HDL-C in in LDLR^–/–^ mice. Of note, compared with model mice, treatment with CP plus proUK significantly reduced the body weight and the plasma level of TC, TG and LDL-C, respectively, by 29.9, 63.4, and 49.0% (Figures 3B–D). CP alone reduced plasma TG levels, but had no influence on the body weight, nor on the plasma TC and LDL-C levels in high fat diet-fed mice. On the other hand, no effect was detected for proUK alone on all the endpoints tested in high fat diet-fed mice. In addition, there was no significant difference in the plasma HDL-C levels among the groups (Figure 3E).

### CP Plus proUK Decreases Lipids Levels in the Liver of High Fat Dieted LDLR^–^/^–^ Mice

The biochemical analysis revealed that high fat diet increased levels of hepatic TC and TG in LDLR^–^/^–^ mice as well, which were attenuated by CP plus proUK, but not by either of the two alone (Figures 4A,B). Consistently, the results of transmission electron microscopy showed less lipid droplets and normal ultrastructure in the livers of CP plus proUK treated mice as compared with model (Figure 4C). In addition, ELISA revealed no significant differences in plasma ALT and AST levels among all groups (Figures 4D,E). Taken together, the results suggested that CP plus proUK decreased hepatic lipids while had no impact on the structure and function of mice liver.

### CP Plus proUK Modulates the Lipid Metabolism-Associated Protein Levels in the Liver of High Fat Diet Fed LDLR^–^/^–^ Mice

In order to explore the underlying mechanism by which CP plus proUK exerted protective effect against atherosclerosis, we further detected the proteins levels that are associated with cholesterol metabolism in the liver. Western blot (Figure 5A) revealed that CP alone or CP plus proUK treatment upregulated the protein levels of ABCG5 (Figure 5G) and ABCG8 (Figure 5H), which participate in hepatic cholesterol efflux to bile. However, no effect was detected for other proteins tested, such as CD36 (Figure 5B), SR-A (Figure 5C), SR-BI (Figure 5D), ABCA1 (Figure 5I), ABCG1 (Figure 5J), CYP7A1, a rate-limiting enzyme for bile acid synthesis (Figure 5F) and HMG-CoA reductase, the rate-limiting enzyme in cholesterol synthesis) (Figure 5E). The levels of proteins (Figure 6A) associated with triglyceride metabolism were evaluated in different groups as well, revealing a significant increase of ATGL (Figure 6B), a protein that participates in triglyceride lipolysis, in the livers of the CP plus proUK group compared with model, whereas other proteins related to triglyceride metabolism, such as HSL (Figure 6C) and PPARα (Figure 6D), did not significantly change among groups.

These results indicated that CP plus proUK increased hepatic cholesterol efflux to bile and triglyceride lipolysis, resulting in decreased TC and TG in the liver and plasma.

### CP Plus proUK Reduces Lipid Accumulation by Modulating the Expression of the Proteins Involved in Lipid Metabolism in Aorta of LDLR^–/–^ Mice

In order to observe changes in lipid accumulation caused by CP or/and proUK treatment, we cultured RAW264.7 macrophages in RPIM 1640 medium containing 10% FBS where in 50 μg/ml ox-LDL or 50 μg/ml Dil ox-LDL was added (Supplementary Method). As shown in Figure 7A, treatment with CP plus proUK significantly reduced lipid droplets in Dil ox-LDL incubated-macrophages. Similarly, CP or/with proUK treatment also attenuated TC and TG accumulation in macrophages (Figures 7B,C). Also, we tested the cholesterol efflux by using NBD-cholesterol (a fluorescent analog of cholesterol), the result showed that cholesterol efflux was similar in each group (Supplementary Figure S4). Also, ROS levels in RAW 264.7 had no difference in each group (Supplementary Figure S3).

The lipid homeostasis in macrophages depends on the balance of influx and efflux of lipid. We thus assessed first the expression of CD36 and SR-A, the two proteins that mediate the phagocytosis in macrophage, in different groups. The result showed a significantly increased expression of the two proteins in high fat diet fed LDLR^–^/^–^ mice compared with chow diet control, which, however, was noticeably attenuated by treatment with CP plus proUK, but not by either of the two alone (Figures 7D,F,G). In addition, ATGL, which mediates neutral cholesterol ester hydrolysis, was upregulated in CP plus proUK group compared with model group (Figures 7D,H). Because the free cholesterol hydrolyzed from cholesterol ester transported by ATP-binding cassette A1 (ABCA1), we thus detected the expression of ABCA1but revealing no difference between model group and CP plus proUK group (Figure 7E).

### CP Plus proUK Inhibits Systemic Inflammation in LDLR^–/–^ Mice

**FIGURE 8:**
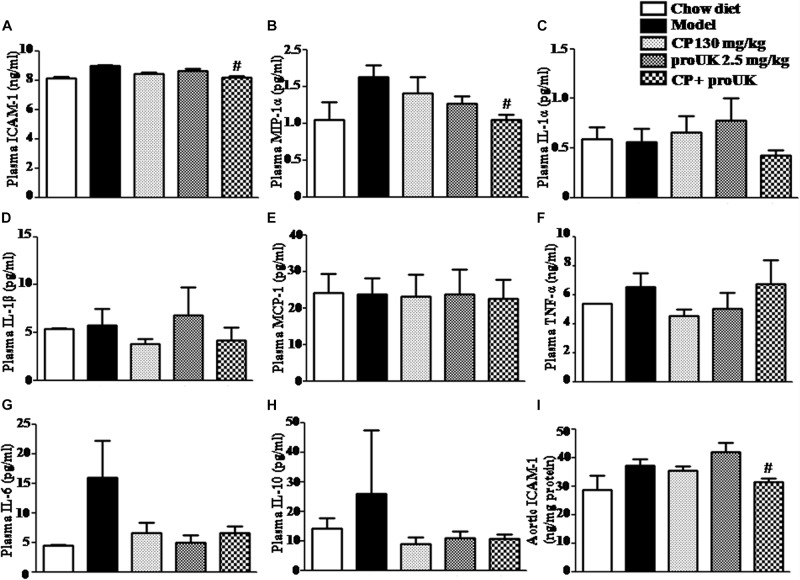
Effect of CP plus proUK on plasma inflammatory cytokine levels in LDLR^–/–^ mice. **(A)** Plasma ICAM-1 detected by ELISA in LDLR^–/–^ mice from different groups. **(B)** Plasma MIP-1α in LDLR^–/–^ mice from various groups. **(C)** Plasma IL-α in LDLR^–/–^ mice from various groups. **(D)** Plasma IL-1β in LDLR^–/–^ mice from various groups. **(E)** Plasma MCP-1 in LDLR^–/–^ mice from various groups. **(F)** Plasma TNF-α in LDLR^–/–^ mice from various groups. **(G)** Plasma IL-6 in LDLR^–/–^ mice from various groups. **(H)** Plasma IL-10 in LDLR^–/–^ mice from various groups. **(B–H)** All the data were acquired by Flow cytometer. **(I)** The concentrations of aortic ICAM-1 protein measured by ELISA in LDLR^–/–^ mice from various groups. The values are presented as the means ± SEM, *n* = 8–12. ^#^*P* < 0.05 vs. Model mice.

We also explored the effect of CP plus proUK on chronic systemic inflammation in LDLR^–/–^ mice. Flow cytometry and ELISA analysis showed that the concentration of ICAM-1 (Figure 8A) and MIP-1α (Figure 8B) was significantly lower in the CP plus proUK group compared with the model group, whereas the levels of the other cytokines (IL-1α, IL-1β, IL-6, IL-10, TNF-α, and MCP-1) and MDA detected did not vary significantly among groups (Figures 8C–H and Supplementary Figure S2). Furthermore, we collected the aorta from LDLR^–/–^ mice and assessed the aortic ICAM-1 levels by ELISA revealing that CP plus proUK significantly decreased aortic ICAM-1 levels (Figure 8I), whereas CP or proUK alone did not affect the aortic ICAM-1 levels. Also, CP plus proUK reduced aortic CD68 levels (Supplementary Figure S1).

## Discussion

The present study provides evidence that CP plus proUK protects against the high fat diet-induced atherosclerosis development in LDLR^–/–^ mice, manifested reduced the areas of atherosclerotic lesions by 34.8%, along with decreased plasma TC, TG, and LDL-C, as well as a reduction in body weight. The beneficial role of CP plus proUK observed is likely mediated by several pathways, as shown by the modulation of the expression of ABCG5, ABCG8, and ATGL in liver, decreased aortic SR-A and CD36 protein levels, and reduced plasma ICAM-1 and MIP-1α after CP plus proUK treatment. Atherosclerosis is a complex pathological process which has not been fully elucidated until now. Previous intervention studies have focused on reducing lipid accumulation and inflammation. Current treatment of atherosclerosis includes statins and antiplatelet drugs, but the clinical feasibility is not completely satisfactory due to the side effects of these medicines. Abnormal lipid metabolism plays a critical role in the occurrence and development of atherosclerosis. An excessive plasma concentration of LDL-C is widely recognized as a causal factor of endothelial dysfunction and atherosclerotic vascular disease (Hartley et al., 2018). CP has been widely applied for prevention and treatment of angina. On the other hand, increasing evidence suggests that CP may interfere in lipid metabolism and pathogenesis of atherosclerosis. To this end, *S. miltiorrhiza*, a main component of CP, was reported to have lipid-lowering activity (Lim et al., 2017), but the underlying mechanism has not been explored yet. It has been reported that CP significantly inhibited the formation of thrombosis formation and platelet function in high fat diet-fed dogs (Zhang et al., 2006; Wang et al., 2014). Niu et al. (2000) showed that Tanshinone II-A, an ingredient of *S. miltiorrhiza*, inhibits low density lipoprotein oxidation *in vitro*. In a high cholesterol feeding rabbit model, Chen et al. (2006) reported that *S. miltiorrhiza* significantly inhibits intimal hyperlipidemia and improved Ass. In the present study, treatment with CP alone for 4 weeks decreased plasma TG level while increased ABCG8 expression in high fat diet fed LDLR^–/–^ mice, consistent with the reports above. However, CP alone did not show any significant effect on the progression of atherosclerosis, nor on other parameters evaluated. These results reflect the complexity of atherosclerosis pathogenesis, to cope with which more strategy should be included in addition to lowering plasma lipids. proUK preferentially activates plasminogen on the fibrin surface, induces fibrin-selective clot lysis and thus is used as a thrombolytic agent. On the other hand, increasing evidence suggests the potential of proUK to interfere in the progression of atherosclerosis. Fibrin is known as a consistent component of human atherosclerotic plaque and it may contribute to plaque growth by stimulation of cell proliferation and by the binding and accumulation of low density lipoprotein (Thompson and Smith, 1989). It is thus anticipated that fibrin hydrolysis by proUK may help impede the progression of atherosclerosis. In addition, study showed that uPA plays a pivotal role in the regulation of cell adhesion, migration, and proliferation during tissue remodeling including plaque formation (Tkachuk et al., 2009). Despite these, to our knowledge, no study is reported as to the role of proUK in protection of atherosclerosis progression. In the present study, we found no effect of proUK alone on the atherosclerosis plaque area in high fat diet fed LDLR^–/–^ mice, nor on other endpoints tested. However, treatment with proUK in combination of CP surprisingly revealed a significantly protective effect on plaque formation and plasma lipid. Of notice, for most of the variables tested, CP plus proUK is more effective than either one of the two alone. On the other hand, for some variables such as liver TC and liver TG, ABSG5, ATGL, SR-A, Plasma MIP-1α, and aortic ICAM-1, only CP plus proUK exhibited effect while either of the two alone showed no effect, suggesting they may have a synergistic effect. This result highlights CP plus proUK as a potential combination in protection of atherosclerosis progression.

Studies were further conducted to gain insight into the possible pathways that mediate the role of CP plus proUK. The results suggested that at least three pathways are implicated in the effect of CP plus proUK. (1) Lipid metabolism, which was evidenced by the finding that ABCG5 and ABCG8, the proteins that promote hepatic cholesterol efflux to bile (Graf et al., 2002; Repa et al., 2002; Yu et al., 2002, 2005), increased after CP plus proUK treatment, and that the expression of aortic ATGL was elevated by CP plus proUK, which participate in triglyceride lipolysis. It is likely that it is the ABCG5, ABCG8, and ATGL work coordinately that lower the plasma lipid lever. (2) Development of foam cells – We found an reduced lipid droplets in RAW264.7 macrophages, and a decreased expression of CD36and SR-A after CP plus proUK treatment, which belong to the scavenger receptor family and mediate the uptake of modified LDLs by macrophages thus take part in the development of foam cells (de Winther et al., 1999; Herijgers et al., 2000). (3) Inflammation – We observed a reduced expression of pro inflammatory cytokines ICAM-1 and MIP-1αby CP plus proUK compared with high fat diet fed LDLR^–/–^ mice, implying involvement of anti-inflammation in the effect of CP plus proUK treatment. Whether or not other pathway (s) are implicated in the effect of CP plus proUK requires further study. Nevertheless, plasma lipid, foam cells, and inflammation are recognized as the major contributors to the formation of atherosclerosis plaque. CP plus proUK exerts effect through the three pathways suggesting this combination to be a multi targeting strategy. However, more study is required to elucidate how these two medicines orchestrate to activate or strength these pathways and what is the contribution of each of the ingredient contained in CP to the lipid lowering effect.

## Conclusion

The present study showed that CP plus proUK significantly ameliorated the development of atherosclerosis, this effect was associated with modulation of plasma lipids, prevention of the development of foam cells and protection of inflammation in high fat diet-fed mice. The results of the present study suggest CP plus proUK as a novel strategy for preventing AS progression. Nevertheless, more study is needed for clinic translation of the present study results, especially the study on whether or not they have adverse side effect.

## Ethics Statement

All surgical procedures performed on animals were approved by Peking University Biomedical Ethics Committee Experimental Animal Ethics Branch (LA2010001). Procedures involving animals and their care were conducted in conformity with international and national law and policies (EU Directive 2010/63/EU for animal experiments, ARRIVE guidelines and the Basel declaration including the 3R concept).

## Author Contributions

J-ND and J-YH designed the experiments. J-ND, KS, C-SP, B-HH, XC, HL, and GL carried out the experiments. J-ND and QL analyzed the experimental results and data. J-ND, QL, J-YF, and J-YH wrote the manuscript.

## Conflict of Interest Statement

The authors declare that the research was conducted in the absence of any commercial or financial relationships that could be construed as a potential conflict of interest.
