# Supplementary material for: Cardiotonic Pills Plus Recombinant Human Prourokinase Ameliorates Atherosclerotic Lesions in LDLR–/– Mice
Source: Front Physiol. 2019 Sep 10;10:1128. doi: 10.3389/fphys.2019.01128 (PMC6747059; doi:10.3389/fphys.2019.01128)
Supplement: Supplementary file 1 [file Table_1.DOCX]

**Supplementary data**

1. **Supplementary method**
   1. Cholesterol efflux analyses

RAW264.7 macrophages were incubated with 5 μg/ml NBD-cholesterol (a fluorescent analogue of cholesterol) (Invitrogen, California, USA ) diluted in RPMI 1640 medium. After 24 hours, washed 3 times with medium. Then, these cells were incubated for 4 hours without or with 10 μg/ml apolipoprotein A-1 and CP/proUK diluted in medium. These cells were lysed with lysis buffer containing 0.1% triton X-100. 150μl medium were isolated and centrifuged at 300g for 10 minutes to pellet any floating cells. The fluorescence intensity was read by Biotek Synergy Hi, set at an excitation wave 470 nm and emission wave 530 nm. The percentage of cholesterol efflux was calculated as a ratio of the fluorescence intensity in the medium against the total fluorescence within cells.

**1.2** ROS levels in RAW264.7 macrophages

RAW264.7 macrophages were incubated in RPMI 1640 medium containing 50 μg/ml ox-LDL, with or without CP, proUK, or CP plus proUK. After 24 hours, these cells were lysed with RIPA lysis buffer (Applygen Technologies Inc, Beijing, China). ROS levels of RAW264.7 macrophages were measured by enzyme-linked immunosorbent assay using an ELISA kit (Andygene, beijing, China), according the instruction of the manufacture. The color absorbance at 450 nm was measured using a Bio-Rad microplate reader.

1. **Supplement figures**


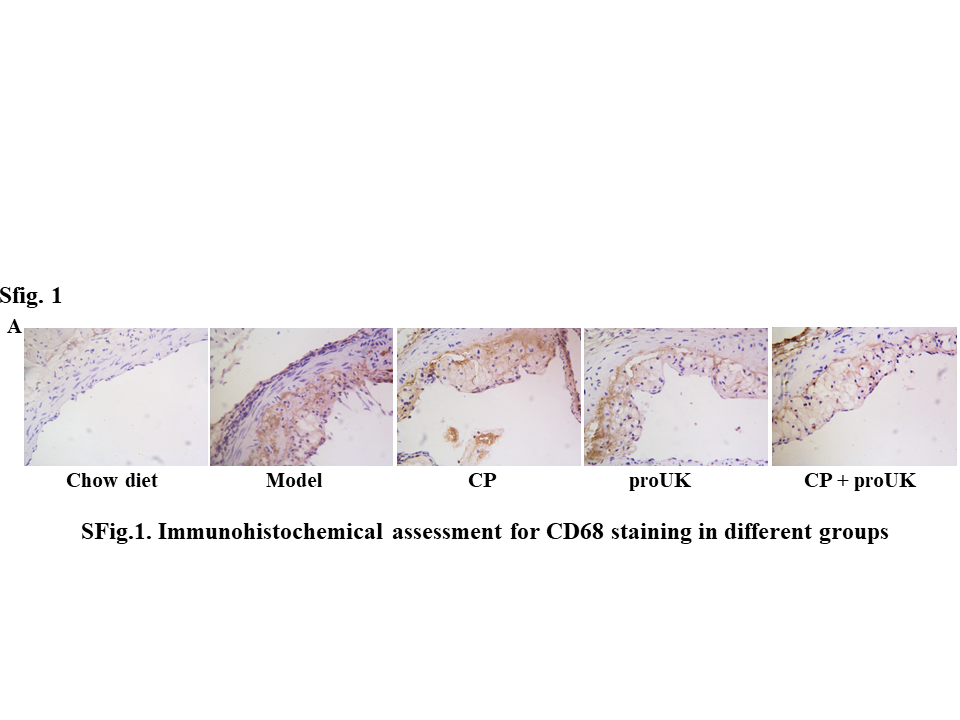


**SFig.1** Representative immunohistochemical assessment of CD68 in the plaque of the left ventricular outflow tract in LDLR-/- mice from Chow diet group, Model group, CP + HFD group, proUK + HFD group, CP + proUK group. N=3

**
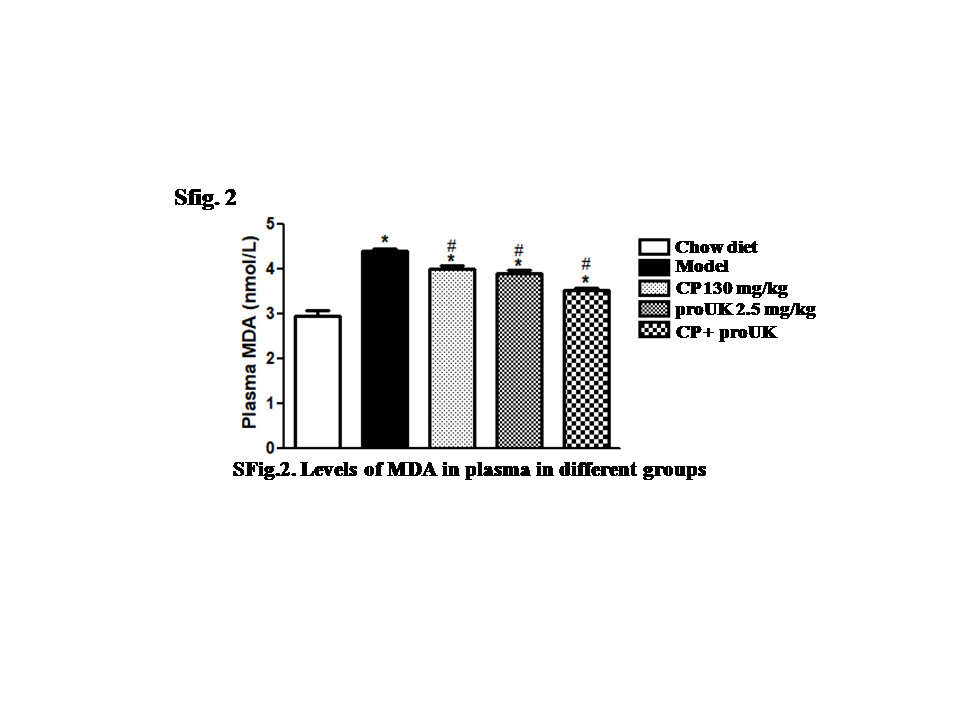
**

**SFig.2** Plasma MDA detected by ELISA in LDLR-/- mice from Chow diet group, Model group, CP + HFD group, proUK + HFD group, CP + proUK group. Data are presented as the mean ± SEM. n=6. *P<0.05 vs Chow diet mice, **^#^**P<0.05 vs Model mice.


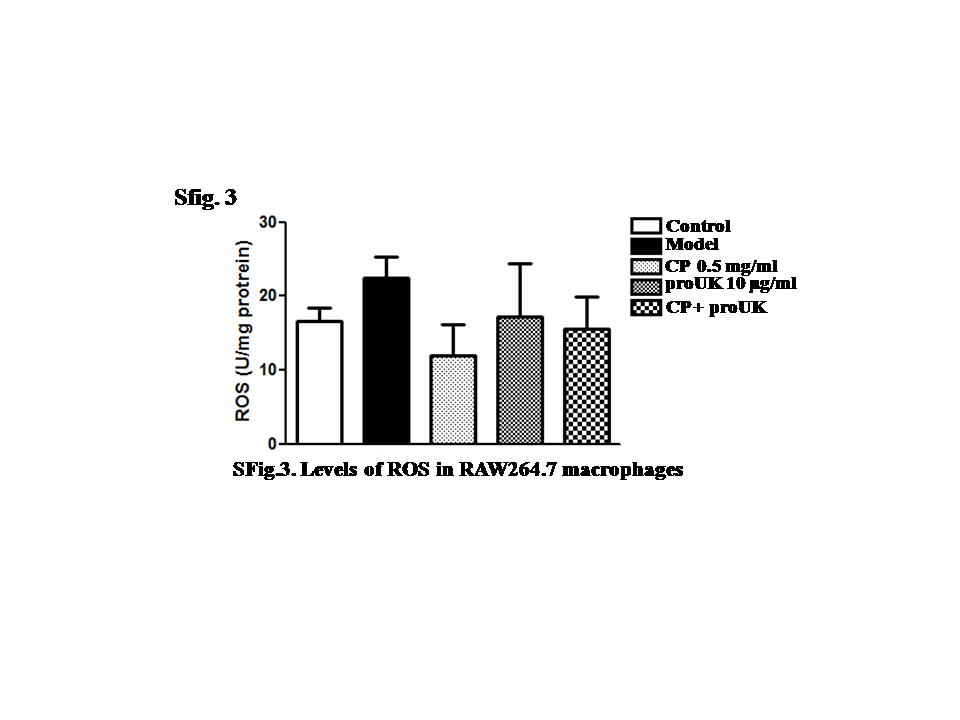


**SFig.3** The level ofROStested by ELISA in RAW264.7 macrophages from control group, Model group, CP + ox-LDL group, proUK + ox-LDL group, CP + proUK+ ox-LDL group. Data are presented as the mean ± SEM. n=6. *P<0.05 vs Chow diet mice, **^#^**P<0.05 vs Model mice


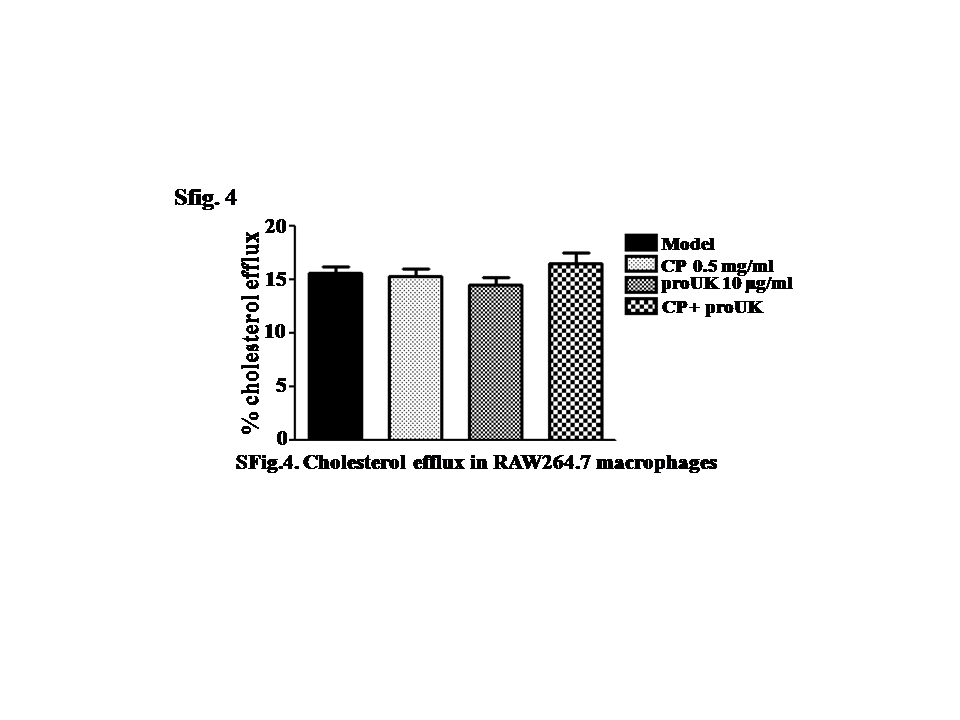


**SFig.4** The level of cholesterol efflux to apolipoprotein A-1 in RAW264.7 macrophages from control group, Model group, CP + ox-LDL group, proUK + ox-LDL group, CP + proUK+ ox-LDL group. Data are presented as the mean ± SEM. n=6.
